# Evaluation of the need for simultaneous orthogonal gated setup imaging

**DOI:** 10.1120/jacmp.v11i2.3203

**Published:** 2010-04-19

**Authors:** Ross I. Berbeco, Seiko Nishioka, Hiroki Shirato

**Affiliations:** ^1^ Department of Radiation Oncology Brigham and Women's Hospital, Dana‐Farber Cancer Institute and Harvard Medical School Boston MA USA; ^2^ Department of Radiation Oncology NTT East‐Sapporo Hospital Sapporo Japan; ^3^ Department of Radiation Medicine Hokkaido University Graduate School of Medicine Sapporo Japan

**Keywords:** respiratory gating, organ motion, IGRT

## Abstract

Image‐guided patient setup for respiratory‐gated radiotherapy often relies on a pair of respiratory‐gated orthogonal radiographs, acquired one after the other. This study quantifies the error due to changes in the internal/external correlation which may affect asynchronous (non‐simultaneous) imaging. The dataset from eight patients includes internal and external coordinates acquired at 30Hz during multi‐fraction SBRT treatments using the Mitsubishi RTRT system coupled with an external surrogate gating device. We performed a computational simulation of the position of an implanted fiducial marker in an asynchronous orthogonal image set. A comparison is made to the reference position, the actual 3D fiducial location at the initial time point, as would be obtainable by simultaneous orthogonal setup imaging at that time point. The time interval between the two simulated radiographic acquisitions was set to a minimum of 30, 60 or 90 seconds, based on our clinical experience. The setup position is derived from a combination of both the initial (AP) and the final (LR) simulated 2D images in the following way: ${\text{LR}}_{\text{setup}}={\text{LR}}_{\text{initial}},{\text{SI}}_{\text{setup}}={\text{SI}}_{\text{initial}}+({\text{SI}}_{\text{final}}-{\text{SI}}_{\text{initial}})/2,{\text{AP}}_{\text{setup}}={\text{AP}}_{\text{final}}$. The 3D error is then the magnitude of the vector from the initial (reference) position to the setup position. The calculation was done for every exhale phase in the data for which there was another one at least 30, 60 or 90 seconds later, at an amplitude within 0.5 mm from the first. A correlation between the time interval and the 3D error was also sought. The mean 3D error is found to be roughly equivalent for time intervals $\left(t_{\text{interval}}\right)$ of 30, 60 and 90 seconds between the orthogonal simulated images (0.8 mm, 0.8 mm, 0.6 mm, respectively). The 3D error is less than 1, 2 and 3 mm for 77%, 89% and 98% of the data points, respectively. The actual time between simulated images turned out to be very close to $t_{\text{interval}}$, with 90% of the second simulated image acquisitions being completed within 38, 68 and 95 seconds of the first simulated image for $t_{\text{interval}}$ of 30, 60 and 90 seconds, respectively. No correlation was found between the length of the time interval and the 3D error. When acquiring respiratory‐gated radiographs for patient setup, only small errors should be expected if those images are not taken simultaneously.

PACS number: 87.55.ne

## I. INTRODUCTION

For a respiratory‐gated treatment, gated imaging should be used for patient setup.^(1)^ The process is similar to a conventional setup; however, the imaging beam (MV or kV) is turned on based on some surrogate of tumor location. The respiratory‐gated image acquisition may be performed manually or automatically, depending on the equipment available. Manual acquisition introduces additional errors due to the variable perception and reflexes of the operator. Ideally, the orthogonal respiratory‐gated setup images should be acquired simultaneously; however, this is not always possible.

Simultaneous, stereoscopic imaging for patient setup has been achieved on several radiotherapy platforms. The CyberKnife system (Accuray Inc., Sunnyvale, CA) uses simultaneous kV imaging from fixed, oblique orientations to localize targets before and during robotic radiosurgery.^(2)^ However, due to the fixed geometry of the imaging, the robotic treatment arm cannot be in certain positions during image acquisition. The Mitsubishi RTRT system (Mitsubishi Medical Systems, Inc., Japan), first introduced in Hokkaido, Japan, offers radiographic and fluoroscopic kV imaging from several oblique angles before and during radiotherapy.^(3)^ Although this system also employs fixed sources and imagers, they are positioned such that imaging may be performed when the linac is at any gantry angle. The Integrated Radiotherapy Imaging System (IRIS) at our institution is capable of acquiring simultaneous orthogonal kV images before and during treatment.^(4)^ ^,^ ^(5)^ The imaging components of the IRIS are mounted to a commercial linac gantry, enabling orthogonal imaging at any gantry position. Any of these systems may be used for simultaneous stereoscopic imaging for patient setup. However, these are all specialized machines and not considered to be appropriate for routine clinical usage in many parts of the world.

Most modern radiotherapy platforms for routine care are equipped with monoscopic kV imaging mounted on the linac gantry, orthogonal to the MV treatment beam.^(6)^ ^,^ ^(7)^ With this configuration, either kV or MV imaging can be used for patient setup, although kV is generally preferable for this purpose due to the higher quality (better contrast) of the images. With either modality (kV or MV), the 3D shift of the patient is estimated from two 2D projections, generally an anterior‐posterior and lateral (left‐right) orthogonal pair. If the same imaging modality is used for each projection, then the linac gantry must be rotated 90° between the orthogonal acquisitions. Alternatively, it may be possible to mix the two modalities (kV and MV) and take simultaneous or near‐simultaneous images.^(8)^ However, this option is not yet commercially available.

As many clinics would prefer to use a pair of orthogonal kV radiographs for patient setup, it is important to quantify the possible errors that could occur due to the asynchrony (non‐simultaneity) of the acquisitions. There may be localization errors if the orthogonal images are separated in time. Intra‐fractional instability of the internal/external correlation has been noted in a previous study.^(9)^ If the time between orthogonal images becomes long enough that variability is more likely, the internal anatomy may not be in the same position for the second respiratory gated radiograph. We have quantified this error using multi‐fraction internal/external data and simulating the asynchronous image acquisition procedure.

Note that the 3D setup error that we are investigating has nothing to do with the relationship of the patient position relative to the simulated (planned) position. The calculation that we are reporting on is a separate issue, accounting only for the error due to the temporal separation of the two orthogonal respiratory‐gated radiographs. All other errors must be assessed independently.

## II. MATERIALS AND METHODS

### A. Respiratory‐gated orthogonal imaging

For this study, we used the abdominal surface as the surrogate of respiratory motion and assumed an automatically respiratory‐gated image acquisition. This is compatible with the RPM respiratory monitoring system combined with the Trilogy iX treatment platform (both from Varian Medical Systems, Inc., Palo Alto, CA). The scenario which is being simulated is one in which a respiratory‐gated 2D projection image is then followed by another one from an orthogonal perspective. Assuming that the first 2D image is an anterior‐posterior (AP) view (gantry=0°), shifts in the superior‐inferior (SI) and medial‐lateral (LR) directions would be visible. Then the second respiratory‐gated radiograph, from either the left or right lateral direction (90° or 270°) would give the coordinates for SI and AP shifts. From our clinical experience, we estimate that the time between the two respiratory‐gated radiographs is no less than 30 seconds and perhaps as long as 90–100 seconds. Therefore, the analysis described below was performed for minimum time intervals of 30, 60 and 90 seconds between the simulated initial and final orthogonal radiographs.

### B. Patient data

The internal and external motion of patients has been derived from the datasets of eight patients undergoing multi‐fraction SBRT treatments.^(10)^ All of these patients had internal target motion greater than 1 cm. This dataset was acquired using a combination of the Mitsubishi RTRT system and the Anzai 733V respiratory gating system (Anzai Medical Systems) at the NTT Hospital in Sapporo, Japan. The RTRT system recorded the 3D internal coordinates of a fiducial implanted in each patient's lung tumor using stereoscopic kV fluoroscopic imaging at 30 Hz. The accuracy and robustness of this system has been well documented.^(3)^ The patient's external anatomy was monitored with the Anzai 733V respiratory‐gating system which consists of a laser displacement device. (More information about this system can be found in a report by Berbeco et al.^(10)^) The combination of these technologies yields 3D internal motion data coupled with 1D external displacement data. Some information about the patients is listed in Table 1; additional patient details can be found in previously published reports.^(9)^ ^,^ ^(10)^ The length of each dataset ranged from 35 to 270 seconds. The total number of pairs of simulated respiratory gates investigated was 1674, 1192 and 789 for 30, 60 and 90 second time delays, respectively.

**Table 1:** Patient information; Patient 2 was treated twice, at the same site, with two months between treatments. The tumor location is indicated using the common anatomical notation for lung segmentation: S1‐3 is upper lobe; S4‐5 is middle lobe, S6‐10 is lower lobe. (n/a=a mean motion less than 2 mm).

*Patient*	*Gender*	*Age*	*Tumor Pathology*	*Tumor Site*	*Mean Internal Target Motion*		
					*LR*	*(cm) AP*	*SI*
1	F	47	Adenocarcinoma	Rt. S4	n/a	0.5	0.7
2	M	81	Squamous cell carcinoma	Rt. S2b	n/a	0.3	0.7
3	M	61	Small cell lung cancer	Rt. S10	n/a	n/a	0.9
4	M	68	Squamous cell carcinoma	Rt. S6	n/a	n/a	1.1
5	M	85	Adenocarcinoma	Rt. S8	n/a	0.4	1.3
6	M	76	Squamous cell carcinoma	Lt. S3	0.3	0.8	0.8
7	M	58	Adenocarcinoma	Lt. S10	0.3	0.9	1.1
8	M	80	Squamous cell carcinoma	Rt. S10	n/a	0.3	1.7

### C. Simulation of patient setup

**Figure 1:**
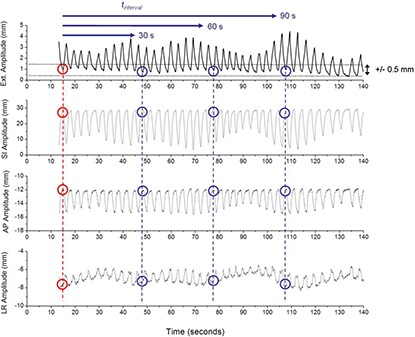
The image acquisition sequence is illustrated. Motion traces for the external surrogate and the target in three dimensions are shown in descending order, respectively. In this example, the initial radiograph is taken at t=15 sec. This initial location of the external surrogate as well as the internal target is shown by the red circles. The final location of the surrogate and target after $t_{interval}>30\;\sec$, 60 sec or 90 sec is shown by the blue circles. The 1 mm gating window for the image acquisition is shown on the uppermost trace.

To simulate respiratory‐gated setup imaging, we used the RTRT data to find the 3D location of the internal marker when the external marker was at end‐of‐exhale, and set this as our true reference position, ${\vec{P}}_{ref}=({\text{LR}}_{\text{ref}},{\text{AP}}_{\text{ref}},{\text{SI}}_{\text{ref}})$. This is the point which would have been reconstructed in 3D by simultaneous radiographs (using IRIS, for example). This is also the position of the internal marker when the first of the two orthogonal 2D radiographs would be acquired. As such, this point also defines ${\vec{P}}_{initial}=(={\vec{P}}_{ref})$. The second internal position, ${\vec{P}}_{final}$, simulating a second simulated setup image from an orthogonal gantry angle, was found by identifying the time point at which the external marker is within the same amplitude gate (±0.5 mm) as for the first simulated acquisition, at least $t_{\text{interval}}$ seconds after the initial time point. The time interval $\left(t_{\text{interval}}\right)$ is set to a minimum of 30, 60 or 90 seconds, respectively; that is, the second acquisition is simulated at the next appropriate end‐of‐exhale after $t_{\text{interval}}$ has passed The simulated setup position is then based on these two simulated images. A diagram illustrating the image acquisition time line of the procedure is shown in Fig. 1.

If the initial image acquisition is simulated with the kV source at 0°, only the LR and SI projection of the position is seen, ${\text{LR}}_{\text{inital}}$ and ${\text{SI}}_{\text{inital}}$. The second simulated image is then acquired from a lateral view, giving the AP and SI positions of the target, ${\text{AP}}_{\text{final}}$ and ${\text{SI}}_{\text{final}}$. The position to which the “patient” is eventually setup is ${\vec{P}}_{setup}$. Since the SI direction is a shared dimension between the two simulated orthogonal asynchronous images, the SI setup position is taken to be halfway between the values found in the initial and final simulated acquisitions.

(1) $${SI}_{setup}={SI}_{initial}+({SI}_{final}-{SI}_{initial})/2$$

The LR and AP positions are found uniquely in the first and second simulated images, respectively: ${\text{LR}}_{\text{setup}}={\text{LR}}_{\text{initial}}$ and ${\text{AP}}_{\text{setup}}={\text{AP}}_{\text{final}}$

The 3D setup error is defined to be $\vec{Error}={\vec{P}}_{setup}-{\vec{P}}_{ref}$ (or, equivalently, ${\vec{P}}_{setup}-{\vec{P}}_{initial}$). Here, ${\vec{P}}_{initial}$ is the 3D location of the fiducial when the initial simulated 2D radiograph is acquired; but also ${\vec{P}}_{initial}={\vec{P}}_{ref}$, the 3D location of the fiducial as determined by a simulated simultaneous orthogonal radiograph acquisition. Therefore, the error in each direction is defined as follows:

(2) $${Error}_{LR}={LR}_{setup}-{LR}_{ref}={LR}_{setup}-{LR}_{initial}$$

(3) $${Error}_{SI}={SI}_{setup}-{SI}_{ref}={SI}_{setup}-{SI}_{initial}$$

(4) $${Error}_{AP}={AP}_{setup}-{AP}_{ref}={AP}_{setup}-{AP}_{initial}$$

Note that the ${\text{Error}}_{\text{LR}}$ equals zero if the initial kV radiograph is taken from the AP direction (kV source=0°). Therefore, the assumption of the simulated imaging order (AP then LR) will bias our results somewhat. However, from our experience with this dataset, there is little expectation of significant deviation in the lateral direction. Therefore, by maximizing the error from changes in the AP motion, we have essentially made this a worst‐case scenario assessment.

The 3D error is the magnitude of the vector from the reference (=initial) position to the setup position:

(5) $$\begin{array}{l} 3D\;\text{Setup}\;\text{Error}=\left|{\vec{P}}_{setup}-{\vec{P}}_{ref}\right|=\\ \;=\left|{\vec{P}}_{setup}-{\vec{P}}_{initial}\right|=\sqrt{{\left(LR_{setup}-LR_{inital}\right)}^{2}+{\left(SI_{setup}-SI_{inital}\right)}^{2}+{\left(AP_{setup}-AP_{inital}\right)}^{2}} \end{array}$$

The calculation was done for every exhale phase for which there was another one at least $t_{\text{interval}}$ seconds later at an amplitude within 0.5 mm of the first. To avoid obviously aberrant end‐of‐exhale locations, an additional restriction to consider only those exhale data points within 2 mm of the mean exhale position is used for the simulation. These would most likely be excluded by the user during an actual image acquisition. In addition, a correlation was sought between the time interval $\left(t_{\text{interval}}\right)$ and the 3D error calculation – the hypothesis being that the larger the interval, the larger the error would be.

## III. RESULTS

**Figure 2:**
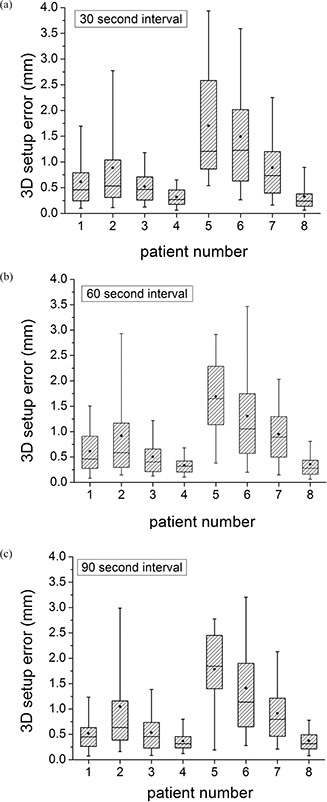
The 3D setup error per patient for $t_{interval}>30\;\sec$ (a), 60 sec (b), and 90 sec (c). The dot represents the mean value, the horizontal line is the median, the shaded box is the 25%–75% range, and the “whiskers” show the 5%–95% range.

The patient‐specific setup error calculations are shown in Figs. 2(a)–2(c) for the three time intervals investigated (30, 60 and 90 seconds). In all of the histogram box plots shown, the dot represents the mean value, the horizontal line is the median, the shaded box is the 25%–75% range, and the “whiskers” show the 5%–95% range. The results for each patient are very similar as the time interval changes. There is some variation from patient to patient; however, the mean for every patient is below 2 mm. Patient 5 has the largest deviations with a mean 3D setup error of 1.7 mm and a 5–95% range of 0.5–3.9 mm $\left(t_{\text{interval}}=30\right)$. Patient 4 has the smallest deviations with a mean 3D setup error of 0.3 mm and a 5–95% range of 0.0–0.6 mm $\left(t_{\text{interval}}=30\right)$.

**Figure 3:**
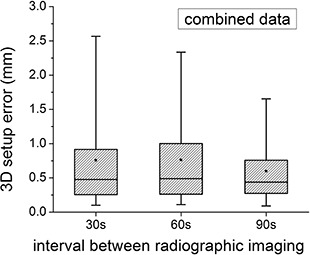
Combined plot of the 3D setup error. The dot represents the mean value, the horizontal line is the median, the shaded box is the 25%–75% range, and the “whiskers” show the 5%–95% range.

Pooling all of the patient data together (Fig. 3), the mean 3D error is found to be equivalent for time intervals of 30, 60 and 90 seconds between the simulated orthogonal images (0.8 mm, 0.8 mm, 0.6 mm, respectively). The 3D error is less than 1, 2 and 3 mm for 77%, 89% and 98% of the data points, respectively. The 3D error is less than 1, 2 and 3 mm for 77%, 89% and 98% of the data points, respectively. The actual time between simulated images turned out to be very close to $t_{\text{interva}}$1, with 90% of the second simulated image acquisitions being completed within 38, 68 and 95 seconds of the first simulated image for $t_{\text{interval}}$ of 30, 60 and 90 seconds, respectively. No correlation was found between the length of the time interval and the 3D error (R<0.3).

**Figure 4:**
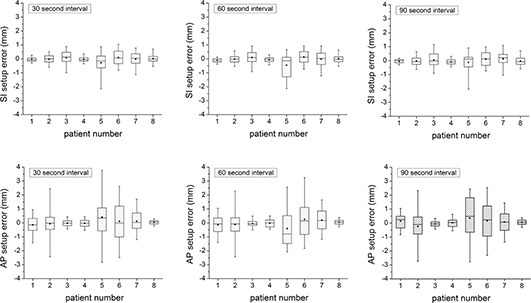
The setup error, broken into SI (top) and AP (bottom) components, for $t_{interval}>30\;\sec$, 60 sec and 90 sec. The dot represents the mean value, the horizontal line is the median, the shaded box is the 25%–75% range, and the “whiskers” show the 5%–95% range.

**Figure 5:**
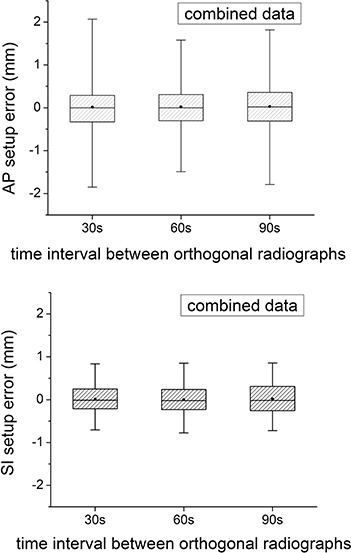
The setup error combined for all the patients in the AP (a) and SI (b) directions, respectively. The dot represents the mean value, the horizontal line is the median, the shaded box is the 25%–75% range, and the “whiskers” show the 5%–95% range.

The 3D error may also be broken down into the individual components (${\text{Error}}_{\text{AP}}$ and ${\text{Error}}_{\text{SI}}$). These results are shown in Fig. 4. Again, the error does not depend significantly on the time interval. Although all errors are close to zero mean, the range of error is smaller in the SI direction than in the AP direction. The average range encompassing 90% of the data in the SI direction only is 1.3 mm, and in the AP direction only is 2.3 mm $\left(t_{\text{interval}}=30\right)$. This becomes more apparent when the patients are combined (Fig. 5). The mean overall setup error in the AP direction is 0.0 with a 5%–95% range from −1.8 to 2.0 mm and, in the SI direction, is 0.0 with a 5%–95% range from −0.7 to 0.8 mm. Considering the SI setup error alone, 99.4% of the data is less than 2 mm from zero.

## IV. DISCUSSION

For the scenario described above, the setup errors associated with asynchronous orthogonal respiratory‐gated kV imaging should not be significant. The majority of the error was found to be in the AP direction. This is most likely due to the fact that the SI error is determined using both radiographic images, essentially halving the error that one would have if only the SI coordinate from the first radiograph was used for the setup. Additional reasons for setup errors in either direction include the size of the setup gating window (1 mm), the relative extent of motion in each direction, and the correlation between the external surrogate and the internal motion in each direction. Details of these last two phenomena were examined in an earlier study.^(9)^ Note that if one wished to minimize the setup error due to asynchronous imaging, as defined in this study, one could image from the lateral (90°/270°) direction first and then from the AP (0o) direction. As always, though, one must account for the other localization errors. Care should be taken to avoid acquiring respiratory‐gated radiographic images during periods of irregular breathing. A method of audio or visual breath coaching may help with this.^(^ ^11^ ^–^ ^13^ ^)^

Previous studies have shown that there is a hardware delay between the external trigger and the resumption of the treatment beam during respiratory gating. This gating delay has been measured to be roughly 0.17 seconds.^(14)^ The analysis described in the current study was reexamined, accounting for the hardware time delay. The difference in the results was found to be negligible (sub‐millimeter).

The formulation of the setup error assumes that common landmarks will be visible in both the initial and final images. Although this may not always be the case in clinical practice, it is unclear how to include this possibility in the analysis that has been presented. No published studies have detailed the frequency of common landmarks in respiratory‐gated images. Therefore, the results of this study do not apply to these cases, as the setup error due to respiratory‐gated imaging, as calculated here, may be an over‐ or underestimation, depending on whether the landmarks are visible in only the first or second image.

The current study focused only on the errors which may be associated with a lengthy respiratory‐gating setup procedure without any consideration of the additional errors which may occur during the treatment delivery. Changes in internal/external correlation or baseline drifts during respiratory‐gated radiotherapy may lead to larger than expected errors in targeted treatment. These phenomena have been reported elsewhere,^(^ ^9^ ^,^ ^15^ ^–^ ^18^ ^)^ and future studies will examine how they may affect the precision and accuracy of respiratory‐gated radiotherapy. Those analyses have been excluded as they are beyond the scope of the work presented here. By focusing on the effect of internal/external correlation variability on asynchronous planar setup imaging only, the results of this work have isolated one part of the respiratory‐gated treatment process and show that no large errors should be expected relative to simultaneous orthogonal respiratory‐gated setup imaging. The intention of this study has been to give confidence to those practitioners of respiratory‐gated radiotherapy who may have been wary of asynchronous setup imaging. The results of this study indicate that it is an acceptable procedure and should not result in additional localization errors to the respiratory‐gated radiotherapy treatment procedure.

## V. CONCLUSIONS

In a study of the errors associated with asynchronous respiratory‐gated setup imaging, it was found that significant errors should not be expected. Other setup and target localization errors were not investigated in this study and should be properly accounted for in the safety margin construction.

## ACKNOWLEDGEMENTS

This project was funded, in part, by a grant from Varian Medical Systems, Inc.
